# ALZpath pTau217: Alzheimer’s disease specificity in the context of multiple comorbidities and predictive capabilities for amyloid burden in combination with other blood‐based biomarkers

**DOI:** 10.1002/alz.093024

**Published:** 2025-01-09

**Authors:** Lauren E Chaby, Jacob Borello, Timothy Vaughan, Stuart D Portbury, Hans Frykman, Anna Mammel, Mary Encarnacion, Geidy E Serrano, Thomas G. Beach, Colin L Masters, Christopher C. Rowe, Christopher J Fowler, Rachael E Wilson, Lianlian Du, Erin M. Jonaitis, Sterling C. Johnson, Andreas Jeromin

**Affiliations:** ^1^ ALZpath, Inc., Carlsbad, CA USA; ^2^ ALZpath, Carlsbad, CA USA; ^3^ BC Neuroimmunology lab, Vancouver, BC Canada; ^4^ Department of Medicine, University of British Columbia, Vancouver, Vancouver, BC Canada; ^5^ Neurocode USA Inc., Bellingham, WA USA; ^6^ Banner Sun Health Research Institute, Sun City, AZ USA; ^7^ ARC Training Centre in Cognitive Computing for Medical Technologies, Parkville, VIC Australia; ^8^ AIBL Research Group, Perth and Melbourne Australia; ^9^ The Florey Institute of Neuroscience and Mental Health, The University of Melbourne, Parkville, VIC Australia; ^10^ Wisconsin Alzheimer's Institute, University of Wisconsin School of Medicine and Public Health, Madison, WI USA; ^11^ University of Wisconsin, Madison, WI USA; ^12^ University of Wisconsin School of Medicine and Public Health, Madison, WI USA

## Abstract

**Background:**

Tau phosphorylated at position 217 (pTau217) is considered to have the highest accuracy in identifying Alzheimer’s disease (AD) pathology using blood. We describe a multi‐cohort evaluation of the Simoa ALZpath pTau217 assay for the prediction of amyloid status in combination with additional blood‐based AD biomarkers (GFAP, pTau181, etc.), as well as comparisons between histopathological and PET based amyloid measurements. We describe the contribution of a spectrum of neurodegenerative diseases and demographic features to circulating plasma pTau217 levels.

**Method:**

The Simoa ALZpath pTau217 assay is an ultra‐sensitive blood‐based assay developed on the semi‐automated single‐molecule array Simoa platform. We evaluate Simoa ALZpath pTau217 in the Banner Brain and Body Donation Program (characterized by several clinically meaningful comorbidities and mid‐to‐late stage AD; pTau217 subset: mean Braak Score = 4.17, ADNC High Plaques = 28%, ADNC Intermediate Plaques = 33%), the Wisconsin Registry for Alzheimer’s Prevention (a longitudinal cohort focused on healthy cognition through MCI), and a cognitively normal amyloid negative subset of the Australian Imaging, Biomarker and Lifestyle (AIBL) study.

**Result:**

We discuss ALZpath pTau217 predictive capabilities in the context of PET imaging and post‐mortem histopathology for total and region‐specific pathological burden and the relative contribution of other blood‐based biomarkers and demographic features. We also report AD and MCI specificity in driving plasma levels of ALZpath pTau217 in the context of multiple comorbidities including CAA, DLB, and VAD.

**Conclusion:**

Findings support ALZpath pTau217 as a high performing biomarker of CNS amyloid and tau burden that is driven specifically by AD and MCI in the context of multiple comorbidities. The ALZpath pTau217 performance capabilities can support timely AD diagnosis and intervention.

